# EPSILON-CP: using deep learning to combine information from multiple sources for protein contact prediction

**DOI:** 10.1186/s12859-017-1713-x

**Published:** 2017-06-17

**Authors:** Kolja Stahl, Michael Schneider, Oliver Brock

**Affiliations:** Robotics and Biology Laboratory, Department of Electrical Engineering and Computer Science, Technische Universität Berlin, Marchstraße 23, Berlin, 10587 Germany

**Keywords:** Contact prediction, Meta algorithms, Deep learning

## Abstract

**Background:**

Accurately predicted contacts allow to compute the 3D structure of a protein. Since the solution space of native residue-residue contact pairs is very large, it is necessary to leverage information to identify relevant regions of the solution space, i.e. correct contacts. Every additional source of information can contribute to narrowing down candidate regions. Therefore, recent methods combined evolutionary and sequence-based information as well as evolutionary and physicochemical information. We develop a new contact predictor (EPSILON-CP) that goes beyond current methods by combining evolutionary, physicochemical, and sequence-based information. The problems resulting from the increased dimensionality and complexity of the learning problem are combated with a careful feature analysis, which results in a drastically reduced feature set. The different information sources are combined using deep neural networks.

**Results:**

On 21 hard CASP11 FM targets, EPSILON-CP achieves a mean precision of 35.7*%* for top- *L*/10 predicted long-range contacts, which is 11% better than the CASP11 winning version of MetaPSICOV. The improvement on 1.5*L* is 17%. Furthermore, in this study we find that the amino acid composition, a commonly used feature, is rendered ineffective in the context of meta approaches. The size of the refined feature set decreased by 75%, enabling a significant increase in training data for machine learning, contributing significantly to the observed improvements.

**Conclusions:**

Exploiting as much and diverse information as possible is key to accurate contact prediction. Simply merging the information introduces new challenges. Our study suggests that critical feature analysis can improve the performance of contact prediction methods that combine multiple information sources. EPSILON-CP is available as a webservice: http://compbio.robotics.tu-berlin.de/epsilon/

**Electronic supplementary material:**

The online version of this article (doi:10.1186/s12859-017-1713-x) contains supplementary material, which is available to authorized users.

## Background

Contact prediction methods identify residue pairs that are in spatial proximity in the native structure of a protein. Contacts can be used as constraints to guide *ab initio* methods [1–5] and to reconstruct the 3D structure of a protein [6–11]. In the 11th Critical Assessment of Structure Prediction (CASP11), a bi-annual set of blind studies to assess the current state of the art in protein structure prediction, predicted contacts were the decisive factor to model the structure of target T0806-D1 with unprecedented accuracy for *ab initio* methods [12]. Furthermore, predicted contacts can be used to compute long-range contact order (LRO) [13] to predict the folding rates of globular, single-domain proteins [14]. This is possible because long-range contact order and its variations correlate with protein folding rates [13, 15–17].

Despite recent successes, contact prediction remains a difficult problem. The difficulty is primarily due to the size of the solution space which renders an exhaustive search unfeasible. To render the search tractable, information are given as priors to constrain the search space. Currently, many different sources of information are used in contact prediction. Each source comes with its specific strengths and weaknesses. It is therefore promising to combine as many sources as possible so as to combine their strengths and to alleviate their weaknesses. Methods from machine learning are well-suited for this task; they can be used to automatically determine how information sources should be combined and which combination is most appropriate under which conditions.

Ensembling is one common approach in machine learning to combine multiple sources of information. It uses diverse models, each capturing different aspects of the data. Ensembling is an established technique to boost the performance of predictors [18–20]. Existing meta methods for contact prediction follow this general idea. They typically outperform methods based on only a single source of information [21–23].

Clearly, merging multiple information sources is a promising way towards improving contact prediction accuracy. However, leveraging multiple sources of information via machine learning introduces new challenges. Inevitably, the combination of information increases the dimensionality of the feature space that is used as input to the machine learning algorithm. This is problematic, because the high dimensionality of the feature space increases learning complexity, data size, and training time. High-dimensional spaces also promote over-fitting because a learner might pick up irrelevant patterns in the data that explains the training data but does not generalize to unseen data. Therefore, we cannot simply rely on the concatenation of features that work well by themselves and need to find a more powerful representation of our information to construct strong prediction methods.

In this paper, we develop a novel meta prediction method called EPSILON-CP (combining **e**volutionary, **p**hysicochemical and **s**equence-based information for **c**ontact **p**rediction, *eps* is extended to epsilon) based on deep neural networks that combines sequence-based, evolutionary, and physicochemical information. In the case of contact prediction, traditional features used in sequence-based methods suffer from high dimensionality. Our study suggests that many of these features are not effective in the context of meta contact prediction. Meta contact predictors include features based on other predictors (for instance co-evolutionary information). We develop a new representation with drastically reduced dimensionality that translates into a deep neural network predictor with improved performance.

We show that this approach reaches 35.7*%* accuracy for the top *L*/10 long-range contacts on 21 CASP11 free modeling target domains, 11% better than the CASP11 winning version of MetaPSICOV, where *L* is the length of the protein. The increase in mean precision on 1.5*L* is 17%. We further show through a feature importance analysis that dropping the amino acid composition from the feature set results in a dimensionality reduction of up to 75%. The approach presented here might be seen as a roadmap to further boost the performance of contact prediction methods.

## Related work

The focus of this paper is the combination of information sources to improve contact prediction. Therefore, we will review related work with respect to the leveraged information sources. In addition, we will discuss how current meta approaches combine multiple information sources for contact prediction.

### Evolutionary information

The first source of information stems from evolutionary methods. Statistical correlations in the mutations of residue pairs are indicative of contacts. Since the mutation of a residue can lead to a destabilization of the structure, the other residue mutates as well to maintain stability. *Evolutionary methods* look for co-evolving patterns in multiple sequence alignments (MSA). Different methods have been developed for the statistical analysis of MSAs and to reduce the effects of phylogenetic bias and transitive couplings that can lead contact prediction astray [24].

Dunn et al. [25] introduced the *average product correction (APC)* to mitigate the effect of phylogenetic bias in the computation of mutual information. PSICOV [26] builds on APC to also remove the effect of indirect coupling. Other approaches work with different assumptions and use pseudo likelihood maximization [27, 28]. The downside of evolutionary methods is that they are critically dependent on the quality of the MSA. The number of homologous sequences in the MSA needs to be in the order of 5*L* sequences [29, 30], where *L* is the length of the protein.

Evolutionary methods are highly specialized. Each method adds only a single dimension (the output of the evolutionary algorithm) to the meta learning approach. They have been shown to work well on their own, but combining multiple different methods can further improve the results [21, 23].

### Sequence-based information

The second source of information is extracted from the sequence of amino acids. Sequence-based contact predictors identify sequence patterns indicative of a contact by applying machine learning on sequence-derived features. SVMcon [31], a sequence-based approach, ranked among the top predictors in CASP7. The approaches vary in their use of machine learning algorithms and the overall composition of the feature set, as well as the training procedures [21, 23, 31–33]. Commonly used features are for example the amino acid composition, secondary structure predictions, and solvent accessibility.

Sequence-based methods are robust when only few sequences are available. Most successful entries in recent CASP experiments had a significant sequence-based component [21, 23, 31]. However, this class of methods does not benefit to the same degree from sequence homologs as evolutionary methods and therefore does not excel even if a large number of sequences is available.

Commonly, sequence-based learners use very high dimensional feature sets with many weak features. An associated problem is the curse of dimensionality. The training data that is necessary for proper generalization increases exponentially [34]. Further, some features may overlap with other more high level features that are used in meta approaches. For example, amino acid compositions or evolutionary profiles identify evolutionary patterns, which is something evolutionary methods do as well.

### Physicochemical information

The third source of information is extracted from candidate structures (decoys). Decoys are the result of sampling the energy function in *ab initio* structure prediction and contain the physicochemical knowledge that is encoded in this function. Since native contacts should be favored by the energy function, they appear more frequently in decoys than non-contacts. A successful approach in CASP9 used simple occurrence statistics [35] to identify contacts. [36] use a similar approach and add and energy-dependent weighting of the decoys. EPC-map [22] uses an intermediate graph structure based on the identified contacts and its neighbors in the decoys to extract additional features.

Structure-based approaches work well in *ab initio* contact prediction because it has lower requirements on the availability of homologous sequences compared to sequence-based approaches [22]. However, *ab initio* structure prediction methods are challenged by large proteins and proteins with complex topology. According to [37], folding simulations become the limiting factor for proteins exceeding 120−150 residues in length. This is again due to the large conformational space that has to be sampled. Further, Rosetta [38] is biased towards low contact order proteins [39].

In the context of meta approaches, physicochemical information might play an important role because it is extracted from structure prediction decoys and not from sequence information. Thus, physicochemical information is orthogonal to sequence-based and evolutionary information.

### Meta approaches

Meta approaches combine multiple sources of information. We will briefly review different meta methods, focusing on what types of information they combine and how they combine the information. The presented methods are categorized based on the combination process into *averaging* and *stacking*. In averaging, the final prediction is a (weighted) average of the contact predictions from multiple methods. In *stacking*, the combination process is treated as a learning problem. The predictions of individual models are used as input features to a machine learning algorithm, usually in combination with other features. Stacking allows to capture more complex relationships in the data than weighted averaging but is also prone to overfitting.

#### Averaging

*EPC-map* [22] combines physicochemical and evolutionary information. The final output is a linear combination of the result of the SVM ensemble, GREMLIN and the frequency *f*_*ij*_ of contact *C*_*ij*_ occurring in the decoys. *MemBrain* [40] combines evolutionary and sequence-based information. The sequence-based approach uses ensembling of models trained on different subsets of the training data. The features are constructed from a window centered at the residue. The final feature vector is created by a) concatenation or b) parallel combination. Additionally, in the latter case, the feature dimensionality is reduced by applying gPCA [41]. The final output is a linear combination of the sequence-based and the evolutionary prediction.

#### Stacking

*BCL::Contact* [42] leverages sequence-based and physicochemical information. The physicochemical information comes from 32 different servers. Each server provides ten different models. Based on these models two features are generated (inverse of minimum distance observed between residues *i*, *j* and how many other servers predicted *i*, *j* to be in contact) and combined with common sequence-based features, all in all 90 features. The classifier is a single layer neural network with 32 neurons.

*PhyCMAP* [43] combines sequence-based information with two co-evolutionary methods (PSICOV, Evfold). The sequence-based features include a context-specific interaction potential and the homologous pairwise contact score. The features (roughly 300) are fused and used to train a Random Forest classifier. Additionally, physical constraints are used to filter false positive predictions.

*PConsC* [21] combines sequence-based information with two different evolutionary methods. The evolutionary information is obtained using different multiple sequence alignments which adds further variety. The final classifier is a Random Forest trained on the fused feature set.

*MetaPSICOV* [23] combines sequence-based information with the output of three evolutionary methods. The high level evolutionary input features are merged with the crude sequence-based features for a total of 672 features. The classifier is a single layer neural network with 50 neurons.

*EPSILON-CP* (our method) uses stacking to combine physicochemical, evolutionary and sequence-based information, therefore extending over current methods. We critically analyze feature importance to reduce the dimensionality of the feature set. As we will show later, this allows us to use a more complex neural network architecture which improves performance (Table 1).

**Table 1:** Overview: Leveraged information per algorithm

Algorithm	Physicochemistry	Evolutionary	Sequence	Features
EPC-map	✓	✓(1)		228
MemBrain		✓(1)	✓	400/200
BCL::Contact	✓		✓	90
PhyCMAP		✓(2)	✓	≈300
PConsC		✓(2)	✓	252
MetaPSICOV		✓(3)	✓	672/731
EPSILON-CP	✓	✓(5)	✓	171

The number of features specified for MetaPSICOV refer to Stage1/ Stage2. The number of features specified for MemBrain refer to the serial/parallel combination. The first part of the table contains methods that linearly combine the features/high-level predictions, the second part non-linearly. The numbers in parentheses for evolutionary information denote the number of different methods utilized

## Methods: Contact Predictor EPSILON-CP

### Contact definition and evaluation

Two residues are considered to be *in contact* if the distance between their respective C _*β*_ atoms (C _*α*_ for glycine) is smaller than 8Å in the native structure of the protein. Contacts are classified according to the sequence separation of the contacting residues. Long-range contacts are separated by at least 24 residues. Long-range contacts capture information about the spatial relationship of parts of the protein that are far apart in the sequence. As a result, they are the most valuable type of contact for structure prediction.

The standard evaluation criteria for contacts is the precision of the top ranked contacts. Predicted contacts are sorted in descending order by confidence and the top predictions up to a given threshold are taken into consideration. We will use *L*/10,*L*/5,*L*/2,*L* and 1.5*L* for the threshold, where *L* is the length of the protein in residues. We use the precision on these lists as the performance criterion. The precision is defined as the ratio of true positives (TP) to the number of true and false positives (FP) combined (Prec = TP / (TP+FP)). True positives are predicted contacts that are indeed in contact in the native structure. False positives are predicted contacts that are not in contact in the native structure.

### Data and training setup

The final neural network is trained on 1542 proteins. The hyperparameters are determined with a 5-fold cross-validation on 1479 proteins, with the remaining 63 proteins as a holdout set. We used random splits for the folds. In total, there are over 22 million training examples. Note that we use 5-fold cross-validation along the different proteins, not contact training examples. This ensures that training samples from validation proteins are unseen by the learner during parameter tuning.

The final training set has been build gradually over intermediate experiments, which are described below. We started from an original training set with 1179 proteins from EPC-map train [22] and MetaPSICOV training sets [23]. To filter similar proteins, we made pairwise comparisons with HHSearch [44] and removed redundant sequences with an E-value below 10^−3^. The E-value is close to zero for a highly specific match (lower is more specific/has higher sequence similarity). In the beginning, we struggled with slow training and could not fit all the data into memory. To alleviate this issue, we subsampled the data. The subsampled training set included 557 proteins from EPC-map train and 187 proteins from MetaPSICOV train that exceeded 200 amino acids (randomly chosen). This set is used in “Feature importance analysis reveals that AA composition is obsolete in meta contact predictors” section. Because of this analysis, we are able to extend the original training set to also include proteins from MetaPSICOV test. These proteins represent difficult prediction cases because they have smaller multiple sequence alignments. To include as many as possible, we relaxed the initial, stringent filtering with HHSearch. We kept 19 proteins that exceed an E-value of 10^−3^ but have a sequence identity below 50% for matches that did not span more than half of the sequence. The mean sequence identity for our whole set is below 77% (computed with ClustalOmega [45]) and the HHsearch E-value is <10^−3^ for roughly 99% of the proteins. This diversity allows for random cross-validation splits without the fear of overfitting.

To further prevent overfitting, we use early stopping on the validation sets. The number of residues range from 25 to 499. The median size of the number of alignments is 377.

We use the following data sets to benchmark our algorithm. The test data sets include proteins from all four CATH [46] classes (mainly alpha, mainly beta, alpha beta and few secondary structures). It covers 31 of the top 100 CATH superfamilies, 178 in total and roughly 50% of all architectures as well as 118 different folds. The mean sequence identity between the proteins from the training set and proteins from the test sets is roughly 6.2*%* (computed with ClustalOmega [45]).

#### CASP11

We take the 21 hard FM targets from CASP11 for which PDB structures are available. The targets are evaluated on a domain basis with the official CASP11 domain assignments (http://predictioncenter.org/casp11/domains_summary.cgi). The sequence lengths are between 110 and 470.

#### NOUMENON

The NOUMENON [47] benchmark data set was designed to avoid observation selection bias in contact prediction. We removed 75 proteins matching a protein in the training set with a HHsearch E-value <10^−3^ to avoid overfitting, leaving 75 proteins.

#### Pooled data set

The last benchmark set pools the proteins from EPC-map_test [22], D329 [48], SVMcon [31] and PSICOV [26]. We removed all proteins with a HHsearch E-value <10^−3^ matching a protein from the training set, leaving a total of 358 proteins. The size of the proteins varies from 46 to 458 amino acids.

#### Data generation

We generate multiple sequence alignments with HHblits [44] (version 2.0.16) with an *E*-value of 10^−3^ and the UniProt20 [49] database from March 2013.

We use the contact prediction scores of the following five evolutionary methods: PSICOV [26], GREMLIN [30], mfDCA [50], CCMpred [27] and GaussDCA [51].

We obtain secondary structure predictions from PSIPRED [52] and solvent accessibility from SOLVPRED.

### Features

Our meta prediction method EPSILON-CP combines sequence-based information, evolutionary information, and physicochemical information. The 171 features are primarily based on the standard sequence-based features that have been used in previous studies [23, 31]. Additionally they include the prediction of five evolutionary methods and EPC-map.

The features can be divided into local and global features. Local features are computed on a window of the sequence. We use two windows of size 9 centered at *i*,*j* and a window of size 5 located at the midway (*i*+*j*)/2, where *i*,*j* denote the sequence position. The column features consist of the secondary structure predictions (probability for H,E,C), solvent accessibility (buried or not buried) and the column entropy of the MSA. All of the column features, in addition to the amino acid composition, are replicated on a global sequence level as well.

The global features consist further of the number of effective sequences in the alignment, the sequence length, number of sequence in the alignments, the sequence positions *i*,*j*, as well as the sequence separation.

The co-evolutionary features are computed for the residue pair i,j. We use the mutual information and the APC corrected mutual information [25] as well as the predictions of PSICOV, GaussDCA, mfDCA, CCMpred and GREMLIN and a mean contact potential [53, 54].

Further, the prediction of EPC-map for *i*,*j* is included. By construction, EPC-map does not have predictions for all possible residue-residue pairs because contacts that do not appear in any decoy are not scored. The absence of an EPC-map prediction is encoded as zero.

### Architecture and training

Our machine learning system is a fully connected 4-hidden layer neural network with 400-200-200-50 neurons. We use the Maxout [55] activation function to model non-linearity and softmax activation in the last layer. Maxout units are generalizations of rectified linear units and have been specifically developed to work in tandem with dropout [56]. Dropout randomly “drops” units and their connections during training. It forces the network to learn a more robust representation and approximately corresponds to training and averaging many smaller networks. This approximation is only accurate for linear layers. Since Maxout units learn an activation function by combining linear pieces, it is linear almost everywhere except at the intersections of the linear pieces. Dropout is therefore more accurate in Maxout networks compared to networks that use non-linear functions [56]. We apply dropout with *p*=0.5 after each hidden layer to avoid overfitting. We use stochastic gradient descent with a learning rate of 0.01, a decay of 1*e* ^−6^ and mini batches of size 100. The gradient descent is accelerated with [57] momentum of 0.5. The weights are initialized using the initialization scheme proposed by [58] which scales the weights by the respective layer dimensions. We set EPC-map predictions on EPC-map_train proteins to zero to avoid overfitting. This is necessary because EPC-map has been trained on EPC-map_train. Each input feature is standardized by subtracting its mean and dividing by standard deviation.

We build the neural network with Keras [59] and trained it between 500 and 600 epochs. Due to dropout, the network can be trained for a long time, both training and validation error still decrease after 500 epochs of training. Training of the neural network was performed on a CPU, not taking advantage of possible speed-ups achievable on a GPU. Training for a single epoch took roughly 45min on a machine with two CPUs and 24 threads. Each CPU has six 2.6GHz cores (E5-2630). The network outputs a probability for each class (contact, non-contact).

We obtained the best results with a first hidden layer that had significantly more units than input features, instead of commonly used bottleneck layers. We hypothesize that this architecture might allow the network to generate a new meta representation that captures of the information more effectively.

## Results and discussion

In the first experiment, we evaluate the performance of EPSILON-CP on the three benchmark sets described in Methods. We compare our method to the CASP11 version of MetaPSICOV [23], which outperformed all other methods in CASP11. At the time of writing, the contact prediction assessment results of CASP12 became available. In CASP12, EPSILON-CP ranked behind the newer version of MetaPSICOV (13th compared to 4th on *L*/5 on the FM targets and long-range contacts and 7th compared to 3rd for long+medium-range contacts).

However, we were not able to perform our own experiments using top CASP12 algorithms because standalone versions of the top servers (RaptorX, iFold, MetaPSICOV) were not available at the time of writing. Our intention in this paper is to control for pipeline effects, such as generated MSAs [23]. Thus, we used the same MSAs as input to all algorithms to measure their performance under the same conditions. RaptorX [60] does provide a web service, but using their pipeline would no longer allow us to control for pipeline effects. As standalone versions of the best CASP12 participating methods are not yet available, we benchmark our algorithm against the best algorithm from CASP11, which is the CASP11 version of MetaPSICOV.

MetaPSICOV operates in two stages. Stage 1 is the output of a neural network classifier. Stage 2 filters predictions of stage 1. We will compare our method to MetaPSICOV stage 2 and will from now on refer to MetaPSICOV stage 2 as MetaPSICOV. Our evaluation focuses on long-range contacts since they are the most helpful in structure prediction. We used the multiple sequence alignments from the original MetaPSICOV paper [23] to ensure that our results are comparable with this study.

In the second experiment, we analyze the importance of features. We evaluate the reduced feature set in the context of meta approaches. To show that the reduction in dimensionality by excluding features is not detrimental to the performance, we compare the performance of the neural network with both the refined feature set and the complete feature set.

In the third experiment we show data that combining multiple sources of information improves the prediction accuracy. We evaluate the performance of the individual methods as well as their combinations. If the assumption holds, performance should improve with additional information sources.

Finally, we briefly discuss limitations of our approach.

### Performance on test data sets

We will first evaluate contact prediction performance on 21 hard free modeling (FM) targets from the CASP11 experiment. Contact prediction is most useful for free modeling targets because they cannot be modeled by only using templates due to the lack of similar structures. In the spirit of the CASP evaluation, we analyzed the results on the basis of the 26 target domains.

**Fig. 1:**
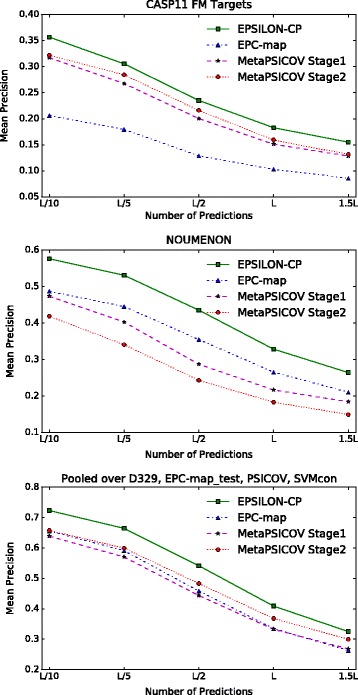
Results for long-range contacts. EPSILON-CP outperforms the other predictors on all three benchmark sets

Figure 1 and Table 2 summarize the performance on the CASP11 set. EPSILON-CP achieves a mean precision of 0.305 on *L*/5, compared to 0.284 for MetaPSICOV. The relative increase in mean precision on average over all cut-offs compared to MetaPSICOV is roughly 12% and 72% over EPC-map. In general, the precision advantage increases with longer cut-offs, see for instance 17% over MetaPSICOV for 1.5*L*. We conducted a paired student t-test to validate the significance of the results. The performance improvement is significant on *L* and 1.5*L*, with a *p*-value <0.022 and 0.001 respectively. In practice, these cut-offs are the most important for structure prediction. Kamisetty et al. [30] observed better performance by using at least *L* contacts, RBO Aleph [61] uses 1.5*L* contacts. For additional comparisons on medium- and long-range contacts to top 10 predictors from CASP11 consult the supplementary section (Additional file 1). It also includes a head-to-head comparison between EPSILON-CP and MetaPSICOV on all 21 FM targets.

**Table 2:** Mean precision for long-range contacts on 21 CASP11 FM hard targets

	L/10	L/5	L/2	L	1.5L
EPC-map	0.206	0.18	0.129	0.103	0.086
MetaPSCIOV (stage 1)	0.317	0.268	0.2	0.151	0.129
MetaPSICOV (stage 2)	0.322	0.284	0.216	0.159	0.132
EPSILON-CP	0.357	0.305	0.235	0.182	0.155
CCMpred	0.221	0.182	0.145	0.111	0.092
GaussDCA	0.209	0.186	0.135	0.104	0.087
GREMLIN	0.207	0.165	0.12	0.086	0.078
PSICOV	0.189	0.147	0.112	0.087	0.074

Precision is computed on the 26 domains of these targets for the top predictions relative to the sequence length *L*

On the NOUMENON [47] dataset the difference in performance is more pronounced. Here, EPSILON-CP outperforms MetaPSICOV stage 2 on average by 65%. The trend continues that the relative mean precision increases over longer cut-offs from 37% on *L*/10 to 77% on 1.5*L*. EPSILON-CP has an accuracy of 26.4*%* on 1.5*L* compared to 14.9*%* for MetaPSICOV stage 2. Notably, MetaPSICOV stage 1 outperforms stage 2 on this dataset. A possible explanation are the low confidence predictions of stage 1 – on this data set even for short-range contacts. Since stage 2 is essentially a filtering step, the performance may further deteriorate because of false negatives.

We also see a strong decline in prediction accuracy for the co-evolutionary methods (see Tables 3 and 4 for comparison). Here, EPSILON-CP outperforms the co-evolutionary methods 5-fold with an accuracy of 53.1*%* on *L*/5 compared to 8.3*%* for GaussDCA. In general, there is a big improvement over the co-evolutionary methods.

**Table 3:** Mean precision for long-range contacts on the NOUMENON data set

	L/10	L/5	L/2	L	1.5L
EPC-map	0.487	0.445	0.355	0.265	0.21
MetaPSCIOV (stage 1)	0.473	0.403	0.287	0.217	0.184
MetaPSCIOV (stage 2)	0.419	0.341	0.243	0.183	0.149
EPSILON-CP	0.576	0.531	0.435	0.328	0.264
CCMpred	0.095	0.095	0.083	0.074	0.066
GaussDCA	0.084	0.083	0.077	0.07	0.065
GREMLIN	0.071	0.073	0.065	0.059	0.056
PSICOV	0.083	0.069	0.063	0.055	0.053

Precision of the top predictions relative to the sequence length *L*

**Table 4:** Mean precision for long-range contacts for proteins from D329, SVMcon Test, PSICOV and EPC-map_test

	L/10	L/5	L/2	L	1.5L
EPC-map	0.656	0.591	0.459	0.335	0.263
MetaPSCIOV (stage 1)	0.639	0.57	0.444	0.333	0.268
MetaPSCIOV (stage 2)	0.658	0.599	0.483	0.368	0.3
EPSILON-CP	0.723	0.665	0.542	0.409	0.325
CCMpred	0.511	0.456	0.345	0.249	0.197
GaussDCA	0.481	0.423	0.322	0.238	0.192
GREMLIN	0.5	0.448	0.338	0.243	0.192
PSICOV	0.452	0.39	0.285	0.203	0.163

Precision of the top predictions relative to the sequence length *L*

On the easier pooled data set the improvements are less pronounced but EPSILON-CP still outperforms the second best method by 10% on *L*/5 improving the accuracy from 59.94*%* to 66.47*%* (see also Table 4 for a complete overview, including results from co-evolutionary methods). The proteins are easier compared to CASP11 and NOUMENON since most proteins have a lot of known homologs.

Summarizing, our classifier improves contact prediction over MetaPSICOV. Further, we could employ a similar strategy to MetaPSICOV stage 2 to boost performance.

### Feature importance analysis reveals that AA composition is obsolete in meta contact predictors

In the previous section, we have shown that our meta prediction method generally improves contact prediction results over MetaPSICOV. Combining multiple sources of information can help to mitigate drawbacks exhibited by individual methods. Similar to ensembling in machine learning, to maximize the impact the sources should be as diverse as possible. However, this approach of combining information as features in a machine learning system also has downsides. The concatenation of features increases the dimensionality of the learning problem which might lead to a harder learning problem and to diminishing returns in prediction precision.

The increase in dimensionality introduces mainly two problems. First, due to the curse of dimensionality the training data that is necessary to generalize correctly increases exponentially [34]. This results in a more complex optimization problem as well as increased data size and slower training. Second, most of the commonly used feature sets have been devised to be used on their own and not in the context of meta approaches. Therefore, the features from different information sources might contain information of the same subspace and thus their combination might not contribute to learning.

To investigate this issue in the context of contact prediction, we re-evaluated the features. In our initial experiments, the training of the neural network suffered especially from the large data set and the high dimensional feature set which lead to slow training. Thus, we conducted a feature analysis to potentially reduce the dimensionality of our learning problem.

Using neural networks for feature selection was not straightforward because there is no simple way of computing feature importance from neural networks and feature selection experiments were unfeasible due to long training times and the amount of features.

Thus, we employed XGBoost [62] for evaluating feature importance, which is a decision tree-based algorithm. During construction, tree-based algorithms perform a feature importance ranking. The feature importance can be used as a starting point to evaluate the feature set. Although interesting, the feature importance sometimes lack meaningfulness. Correlation can inflate or deflate the importance of a feature. XGBoost splits the data set recursively. In each split, the feature that best separates the two classes is chosen. Features used in earlier splits are deemed more important. The specific feature importance measure we use is called mean decrease of impurity [63]. Thus, the feature importance values need to be critically analyzed.

**Fig. 2:**
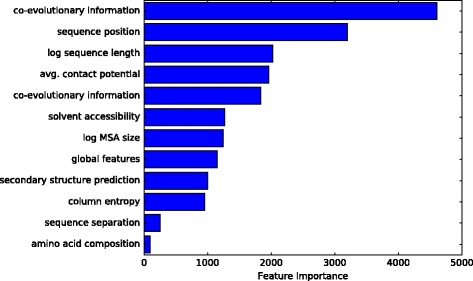
Simplified and aggregated depiction of the feature importance as emitted by XGBoost. The amino acid composition is attributed with the least importance, although it makes up roughly 75% of the features. The different co-evolutionary information entries correspond to different co-evolutionary methods. The feature importance depicted here is the average over a 5-fold cross-validation

An excerpt of the results is shown in the feature importance plot (see Fig. 2, average over 5-fold cross-validation). We include the results of two co-evolutionary methods to show the difference in importance depending on the method. For the purpose of visibility, the rest are left out and some features with fairly similar importance have been aggregated and averaged (see for instance the global features).

Strikingly, the feature importance of the amino acid composition is the lowest of all features. Interestingly, this feature has 485 dimensions and makes up 75% of the features. Note that the real feature importance according to XGBoost might be masked by the aforementioned covariance issues. Nevertheless, we used this feature importance information to refine our representation and re-train the neural network model.

**Fig. 3:**
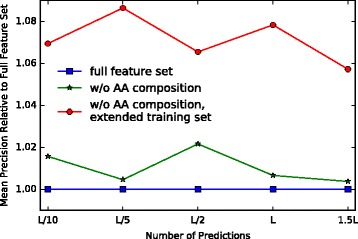
Comparison of three neural networks with identical architecture on EPC-map_test (long-range contacts). The baseline network (square marker) uses the full feature set and is trained on 657 proteins. The training proteins are a mix of EPC-map_train and MetaPSICOV proteins. The square marker denotes the neural network that is trained without the amino acid composition but on the same data set. The second network (circle marker) shows the performance of the neural network after increasing the training set size from 657 to 1479 proteins, which was possible because dropping the amino acid composition reduced the dimensionality of the learning problem. Note here that most of the new proteins are much more complex and may not be helpful for predicting proteins in EPC-map_test

To test the utility of the amino acid composition in our neural network model, we re-trained the system with and without the amino acid composition. The result is depicted in Fig. 3. Removing the amino acid composition does not harm performance. Performance actually increases slightly by 1−2*%*, likely due to the easier optimization problem (see Fig. 3 square and star marker). The results are based on our original training set, a smaller set where the feature set does not yet contain EPC-map. The smaller training set is a strict subset of the final training set, combining 557 proteins from EPC-map_train with 100 randomly chosen proteins from the MetaPSICOV training set that exceed 200 amino acids. Due to the aforementioned scaling issues, we cannot replicate the experiment on the whole data set in a reasonable time with the neural network. With XGBoost however, we were able to verify that the performance is not harmed. XGBoost allowed us to test both configurations (with and without amino acid composition) on the whole data set. The difference in performance is less pronounced because XGBoost already largely ignores unimportant variables, but here the accuracy improved as well or at least remained the same (tested via 5-fold cross-validation). For instance on *L*/10 the accuracy increased from 53.96*%* to 54.57*%*, on medium-range contacts the performance increased on average by 0.5*%*.

Training on more data generally improves the performance of neural networks [64]. The significant reduction in dimensionality made it possible to increase the training set size from the smaller, original training set to the final training set described in **Data and Training Setup**. The performance improvements are depicted in Fig. 3 (circle marker compared to star marker). Here, we compare the performance without the amino acid composition on the original and the final training set. On EPC-map_test the mean precision increased by 6% on long-range contacts.

Our assumption is that the introduction of evolutionary information renders the amino acid composition redundant.

### Combination of different sources of information

**Fig. 4:**
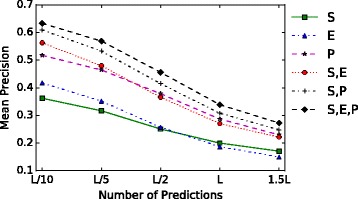
Performance comparison of different information types on long-range contacts on the EPC-map_test data set. S(equence), E(volutionary), P(hysicochemical) and the respective combinations. S uses the feature set described in Features minus EPC-map and the co-evolutionary methods. E (GaussDCA) is the best evolutionary method in our experiments. For P the representative is EPC-map

In this section, we aim to quantify the benefit on contact prediction accuracy of combining multiple information sources. Figure 4 compares the performance of the individual types of information on long-range contacts on the EPC-map_test data set. We use the following abbreviations: S for sequence-based information, E for evolutionary information and P for physicochemical information. S uses the feature set introduced in Features (see “Features” section) without the input features obtained from the co-evolutionary methods and EPC-map. We pick GaussDCA [51] as the representative for evolutionary information because it had the highest feature importance out of all the evolutionary methods in our experiment. For physicochemical information we use EPC-map as the representative algorithm. In this experiment, we removed GREMLIN from the EPC-map algorithm. Strictly speaking, the physicochemical predictor in EPC-map also contains some sequence-based features and could also be seen as a mix of sequence-based and physicochemical information. The EPC-map_test set contains many small proteins with rather small multiple sequence alignments [22]. EPC-map performs well on this set with a mean accuracy of 50% [22] because the small protein size (smaller than 150 amino acids) enables generation of decoys with good quality which impacts physicochemical contact prediction. Nevertheless, combining multiple sources of information clearly improves the results. The performance increases by almost 80% from 31.6*%* on *L*/5 for S to 56.8*%* for S,E,P. The increase over P is 22%.

### Limitations

The main limitation of our approach is that contacts are predicted in isolation. Since there are reoccurring contact patterns, it makes sense to try to incorporate predictions of surrounding contacts. This could be done in a similar fashion to MetaPSICOV stage 2, where an excerpt of the contact map is used as an input for a second model. Ideally, this could be done with end-to-end learning and include a feedback loop.

A second limitation concerns the feature set. Most of the feature set is computed on a fixed window of the sequence potentially ignoring useful information. More powerful methods that work directly on sequences (convolutional neural networks, recurrent neural networks) instead may be able to lift this limitation.

## Conclusion

We presented EPSILON-CP, a contact predictor that combines evolutionary information from multiple sequence alignments with physicochemical information from structure prediction methods and with sequence-based information. These three sources of information are combined using a deep neural network model. We show that combining multiple sources of information improves prediction accuracy when compared to the CASP11 winning version of MetaPSICOV. We use stacking and train a deep neural network to derive on this relationship from data, effectively learning when a specific source of information is most likely to be effective.

The key to performance improvements achieved by our method is the reduced and refined feature set. Due to a careful feature analysis, we found that the amino acid composition, a commonly used feature, can be removed without harming the performance. Our hypothesis is that the introduction of evolutionary methods make the amino acid composition redundant. Our results show that common features must be re-evaluated in the context of meta approaches so as to avoid redundant features that do not contribute to learning. We removed features related to the amino acid composition, reducing the size of the feature set by 75%. This allowed us to train more complex networks and to increase the size of the training set considerably. Using this strategy, EPSILON-CP achieves 35.7*%* mean precision for the top *L*/10 predicted long-range contacts on 21 CASP11 FM hard targets, 11% higher than the second-best method. For the top 1.5*L* long-range contacts the improvement is 17%.

Our study suggests that further improvements in contact prediction will arise from adequately balancing feature-set size and feature expressivity on the one hand, and the size of the training data and the complexity of machine learning algorithms on the other hand. We demonstrated that a reduced feature set enables an increased amount of training data, which leads to improved contact prediction. We hypothesize that further improvements will result from creating even more powerful and compact feature sets that in turn enable the expansion of the training set and the use of more sophisticated learning methods.

## Additional file

Additional file 1 Supplementary. Further comparisons on CASP11 to top 10 predictors on medium- and long-range contacts. Comparison of prediction accuracy for different number of effective sequences as computed in the MetaPSICOV paper [23]. Head-to-head comparison of MetaPSICOV and EPSILON-CP on all 21 FM targets for long-range contacts. (PDF 85.1 kb)

## Abbreviations

AA: Amino acid composition
APC: Average product correction
CASP: Critical assessment of structure prediction
FM: Free modeling
FP: False positives
MSA: Multiple sequence alignments
SGD: Stochastic gradient descent
TP: True positives
