# Supplementary material for: EPSILON-CP: using deep learning to combine information from multiple sources for protein contact prediction
Source: BMC Bioinformatics. 2017 Jun 17;18:303. doi: 10.1186/s12859-017-1713-x (PMC5474060; doi:10.1186/s12859-017-1713-x)
Supplement: Additional file 1 — Supplementary. Further comparisons on CASP11 to top 10 predictors on medium- and long-range contacts. Comparison of prediction accuracy for different number of effective sequences as computed in the MetaPSICOV paper [23]. Head-to-head comparison of MetaPSICOV and EPSILON-CP on all 21 FM targets for long-range contacts. (PDF 85.1 kb) [file 12859_2017_1713_MOESM1_ESM.pdf]

Supplementary

CASP11 results evaluated on the domains of the following 19 FM Targets:
T0761, T0763, T0767, T0771, T0777, T0781, T0785, T0794, T0806, T0808, T0814, T0820, T0824, T0827, T0831, T0832, T0834, T0836, T0855
We had to remove the targets for which we did not have a PDB or predictions of all predictors.

Table 1: Mean precision for medium-range contacts on 19 CASP11 FM targets and a total of 23 domains. Precision of the top predictions relative to the sequence length L.

	L/10	L/5	L/2	L	1.5L
EPC-map	0.517	0.419	0.287	0.196	0.152
MetaPSCIOV (CONSIP2)	0.512	0.418	0.302	0.219	0.171
EPSILON-CP	0.574	0.475	0.329	0.226	0.177
Shen-Group	0.421	0.359	0.256	0.190	0.154
MULTICOM-CLUSTER	0.387	0.340	0.243	0.179	0.143
RaptorX-Contact	0.447	0.368	0.262	0.192	0.156
Pcons-net	0.178	0.157	0.116	0.090	0.077

Table 2: Mean precision for long-range contacts on 19 CASP11 FM targets and a total of 23 domains. Precision of the top predictions relative to the sequence length L.

	L/10	L/5	L/2	L	1.5L
EPC-map	0.181	0.152	0.110	0.090	0.075
MetaPSCIOV (CONSIP2)	0.297	0.269	0.207	0.153	0.126
EPSILON-CP	0.311	0.267	0.211	0.167	0.145
Shen-Group	0.198	0.189	0.153	0.116	0.105
MULTICOM-CLUSTER	0.148	0.117	0.105	0.087	0.082
RaptorX-Contact	0.164	0.143	0.113	0.094	0.086
Pcons-net	0.181	0.140	0.110	0.091	0.077

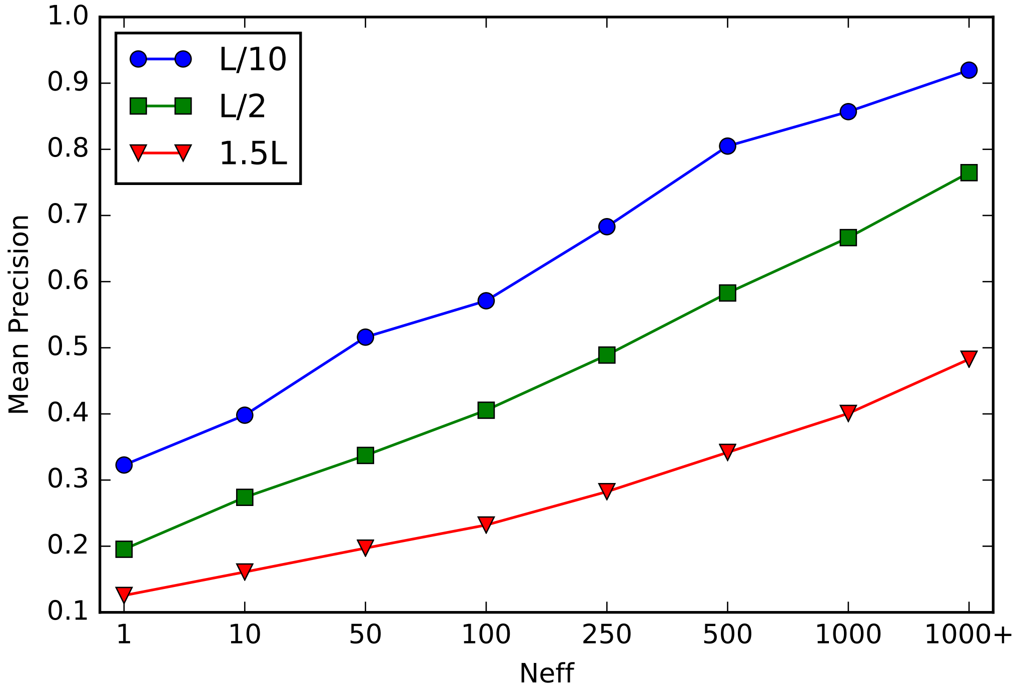

Figure 1: Comparison of the mean precision for different number of effective sequences and different cut-offs tested over all proteins from the benchmark data sets on long-range contacts. The N_{eff} are binned as follows. N_{eff} of 10 includes all proteins with $N_{eff} \in (1, 10]$, 1000+ is $N_{eff} \in (1000, \text{inf}]$. For details of the computation of N_{eff} see "MetaPSICOV: combining coevolution methods for accurate prediction of contacts and long range hydrogen bonding in proteins" by Jones et al.

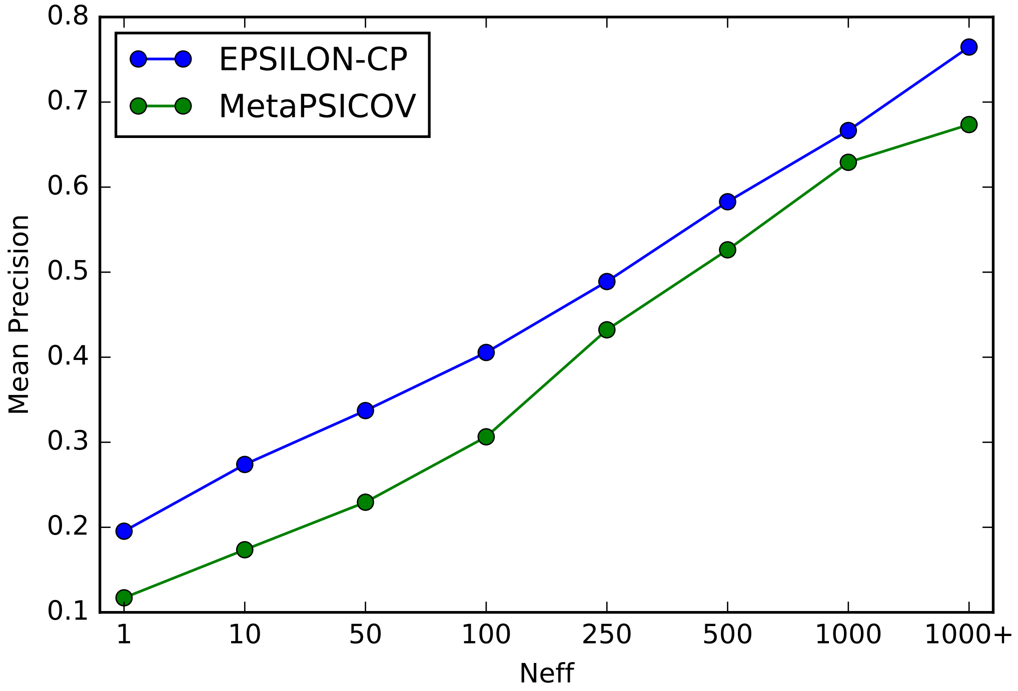

Figure 2: Comparison of the mean precision for different number of effective sequences tested over all proteins from the benchmark data sets for $L/2$ long-range contacts. The N_{eff} are binned as follows. N_{eff} of 10 includes all proteins with $N_{eff} \in (1, 10]$, 1000+ is $N_{eff} \in (1000, \text{inf}]$. For details of the computation of N_{eff} see "MetaPSICOV: combining coevolution methods for accurate prediction of contacts and long range hydrogen bonding in proteins" by Jones et al.

Table 3: Head-to-head comparison of EPSILON-CP and MetaPSICOV on 21 CASP11 FM Targets (long-range contacts).

Target	N_{eff}	MSA size	Method	L/10	L/5	L/2	L	1.5L
**T0761**	1	1	MetaPSCIOV	0.000	**0.024**	**0.019**	0.014	0.022
			EPSILON-CP	0.000	0.000	0.009	**0.019**	**0.028**
**T0763**	2	4	MetaPSCIOV	**0.615**	**0.462**	**0.262**	**0.169**	**0.128**
			EPSILON-CP	0.385	0.231	0.123	0.123	0.092
**T0767**	32	246	MetaPSCIOV	**0.444**	0.352	0.248	0.161	0.136
			EPSILON-CP	0.333	**0.407**	**0.343**	**0.212**	**0.175**
**T0771**	8	15	MetaPSCIOV	**0.118**	**0.114**	0.067	0.079	0.071
			EPSILON-CP	0.059	0.057	**0.112**	**0.096**	**0.086**
**T0777**	36	193	MetaPSCIOV	**0.324**	**0.232**	**0.128**	0.078	0.068
			EPSILON-CP	0.176	0.116	0.105	**0.096**	**0.087**
**T0781**	2	2	MetaPSCIOV	0.237	0.197	0.115	0.081	0.066
			EPSILON-CP	**0.289**	**0.276**	**0.173**	**0.113**	**0.084**
**T0785**	1	1	MetaPSCIOV	**0.182**	**0.182**	**0.125**	**0.134**	**0.125**
			EPSILON-CP	**0.182**	0.091	0.107	0.098	0.107
**T0791**	243	827	MetaPSCIOV	**0.643**	0.509	**0.357**	0.254	0.200
			EPSILON-CP	**0.643**	**0.579**	**0.357**	**0.268**	**0.212**
**T0794**	224	6044	MetaPSCIOV	**0.957**	**0.793**	0.563	0.398	**0.316**
			EPSILON-CP	0.870	0.750	**0.567**	**0.400**	0.310
**T0806**	549	3316	MetaPSCIOV	**0.840**	**0.843**	0.609	0.457	0.370
			EPSILON-CP	0.800	0.765	**0.656**	**0.469**	**0.375**
**T0808**	64	179	MetaPSCIOV	0.225	0.300	0.245	0.198	0.178
			EPSILON-CP	**0.475**	**0.388**	**0.270**	**0.237**	**0.200**
**T0814**	137	352	MetaPSCIOV	0.641	0.506	**0.389**	0.262	0.205
			EPSILON-CP	**0.667**	**0.582**	0.384	**0.277**	**0.230**
**T0820**	1	1	MetaPSCIOV	0.077	0.038	0.015	0.015	0.010
			EPSILON-CP	**0.154**	**0.077**	**0.030**	**0.023**	**0.020**
**T0824**	157	406	MetaPSCIOV	**0.600**	0.476	**0.389**	**0.278**	0.228
			EPSILON-CP	0.500	**0.571**	**0.389**	**0.278**	**0.235**
**T0827**	213	407	MetaPSCIOV	0.500	0.368	0.222	0.163	0.148
			EPSILON-CP	**0.735**	**0.544**	**0.386**	**0.271**	**0.208**
**T0831**	87	281	MetaPSCIOV	0.429	**0.400**	**0.216**	0.136	0.112
			EPSILON-CP	**0.457**	0.343	0.205	**0.153**	**0.121**
**T0832**	10	18	MetaPSCIOV	**0.050**	0.024	0.029	0.043	0.038
			EPSILON-CP	**0.050**	**0.049**	**0.038**	**0.072**	**0.067**
**T0834**	29	160	MetaPSCIOV	0.000	**0.049**	0.048	0.038	0.029
			EPSILON-CP	**0.100**	**0.049**	**0.077**	**0.062**	**0.051**
**T0836**	50	926	MetaPSCIOV	**0.550**	**0.450**	**0.314**	0.167	0.127
			EPSILON-CP	0.450	**0.450**	0.245	**0.181**	**0.144**
**T0837**	9	149	MetaPSCIOV	0.333	0.292	0.183	0.140	0.122
			EPSILON-CP	**0.833**	**0.750**	**0.533**	**0.347**	**0.265**
**T0855**	18	44	MetaPSCIOV	**0.182**	**0.174**	**0.136**	**0.126**	0.107
			EPSILON-CP	**0.182**	0.087	**0.136**	0.118	**0.140**
